# Supplementary material for: Metaplasia of respiratory and digestive tissues in the eastern oyster Crassostrea virginica associated with the Deepwater Horizon oil spill
Source: PLoS One. 2021 Sep 7;16(9):e0247739. doi: 10.1371/journal.pone.0247739 (PMC8423294; doi:10.1371/journal.pone.0247739)
Supplement: Attachment — Submitted filename: Roopnarine_etal_response to reviewers.pdf [file pone.0247739.s001.pdf]

<u>Responses to reviewers</u>
PONE-D-21-04744
Metaplasia of respiratory and digestive tissues in the Eastern oyster *Crassostrea virginica* associated with the Deepwater Horizon oil spill

<u>Reviewer #1</u>
"*Oyster samples were initially placed on ice during sampling and later stored at -18oC to -20oC until histological analysis with storage times varying between 3 days to 12 months (Line 201). Tissues meant for histological analysis are generally stored in 70% alcohol or in formalin. This is because freeing can damage and alter tissues/cells. Expansion of water due to freezing stretches and damages cell membranes. Some of the effects of freezing are loss of epithelial cilia and intracellular vacuolation of cells, which are also some of the reported effects in this manuscript. See Baraibar MA, Schoning P. Effects of freezing and frozen storage on histological characteristics of canine tissues. J Forensic Sci. 1985 Apr;30(2):439-47. The different storage times 3 days-12 months are particularly problematic because the damage caused by freezing increases with duration of freezing (see https://avanan.url-protection.) . If the authors preferred freezing, an alternative would have been fast freezing in liquid nitrogen, and subsequent storage at -80 degrees to avoid formation of tissue-damaging ice crystals. Unless the authors can provide supporting literature to validate their method, this is a fatal error which can seriously affect the findings.*"
We agree with the reviewer and were of this potential problem. As explained in the revised manuscript, the specimens were not originally intended for histological analysis, but instead for chemical analyses. Therefore, soft tissues were not placed in preservatives suitable for histological analysis. Our decision to pursue histological analysis raised the potential issues of freezing, but we addressed this by treating our control specimens from Chesapeake Bay, Maryland, in the same manner as specimens from the Gulf of Mexico, including deep freezing the specimens. Given that those specimens did not exhibit the metaplasia observed in the Gulf of Mexico specimens, we concluded that metaplasia in the latter specimens was not an artifact of freezing. We have explained this in more detail within the manuscript as follows:

Lines 190-198: Freezing as a preservation technique for histological analysis can be problematic because of the formation of ice crystals at the cellular level, and the consequential damage of tissues. Specimens for this study were frozen because they were originally intended for chemical analysis of soft and hard tissues, specifically the measurement of heavy metal concentrations [40]. Thus chemical preservation was avoided and freezing employed instead. We tested for the possible introduction of histological artifacts due to freezing, and the possibility of attributing those incorrectly to metaplasia, by freezing the control specimens from Chesapeake Bay at temperatures and for durations comparable to those used for the GoM specimens.

Lines 248-263: Specimens from the GoM were frozen for periods ranging from one to 16 months (average = 6.2 months), and control specimens from Chesapeake Bay were frozen between one and eight months (average = 2.5 months). We tested for the dependence of the occurrence of any type of metaplasia on the duration for which a specimen was frozen prior to histological analysis, and rejected any such dependence for both ctenidial and digestive tissues (Logistic regression: ctenidia, $\chi2 = 0.25$, $p = 0.617$; digestive, $\chi2 = 1.97$, $p = 0.161$). All GoM specimens exhibited some type of ctenidial metaplasia regardless of the duration of freezing, whereas no specimens from Chesapeake Bay did. GoM specimens exhibited the highest frequency (60%) of digestive tract metaplasia from a single cohort, those collected from Grand Isle in August 2013, and frozen for two

months prior to analysis. Specimens frozen for seven and 15 months comprise the remaining specimens exhibiting digestive tract metaplasia, but metaplasia was absent in specimens frozen for three, seven and 10 months. A single specimen from Chesapeake Bay exhibited digestive tract metaplasia, and it was frozen for three months prior to analysis. We therefore conclude that the duration of freezing prior to analysis did not introduce tissue artifacts that would otherwise bias the following results.

"*The introduction needs to be streamlined and shortened, particularly after page 3.*"
We eliminated sections of the Introduction and shortened it overall, as can be followed in the tracked changes version of the manuscript.

"*Figure 2 are colour bar graphs in red and blue. I suggest these be changed or modified to include patten fill so as to improve accessibility for the visually impaired, particularly the colour blind.*"
We have modified both Figure 2 and the other bar graph figure so that they are no longer coloured, but instead use black, white and grey solid bars. The contrast is therefore increased and colour removed.

"*The micrographs Figure 1,3,4 have a low resolution. It will help improve the quality if the authors have images of higher quality.*"
The micrographs are all of a minimum resolution of 300 dpi (or higher) as required by PLoS, and thus meet the journal's specifications. Several of the higher magnification images were made under oil immersion, perhaps giving the impression that they are of low resolution, but they are not.

"*Line 34: correct disperants to dispersants*"
Corrected.

<u>Reviewer #2</u>
"*The authors also failed to mention if these specimens were of the same size or life stages – different life stages might exhibit different response or degree of metaplasia towards oil spill; and if water parameters were taken during sampling – this could provide a stronger correlation between the present and level of oil in water column with the occurrence of metaplasia.*"
Specimens were of variable size, but all were of sufficient size where it is believed that the species has attained sexual maturity, as outlined in government collecting guidelines. Water samples were unfortunately not taken when specimens were collected, but at no times were oil slicks visible, as mentioned in the Introduction. We have revised the manuscript to describe both shell and soft tissue sizes of the specimens, as follows:

Lines 186-187: Shells ranged in height between 8-12 cm, ensuring that all individuals were post-juveniles.

Lines 208-209: Soft tissue heights ranged between 4-9 cm (average and standard deviation = 6.4±1.26 cm).

"*Since the authors mentioned that metaplasia could be present years after a spill (line 127-138), I would expect the authors to include samples after half a decade and current year as well?*"
Unfortunately this was not possible. Collecting after 2013 became logistically difficult for the research team because no authors were in close proximity to any of the sampling sites. Nevertheless, we planned to take advantage of the extensive collecting undertaken by the United States federal government during that time period. When the specimens were made available to researchers in 2017 we applied for, and received more than 100 specimens from various locations in the Gulf of Mexico.

Unfortunately, none of those specimens had been preserved properly, being mostly dessicated and there unsuitable for histological analysis.

“*Line 140 & 143: ‘et al.’*”
Corrected.

“*Line 239: The authors mentioned in the Method section, only 38 samples were used, not 46?*”
The correct number is 38, and we have made that consistent throughout the manuscript.

“*For all histological figures, please standardize the placement of scale bar to only lower right of each figure.*”
This is not possible. Scale bars are placed automatically by the calibration and imaging software of the microscope’s camera for most of the images, and moving the bar would entail altering/editing the image, which we declined to do.
